# Sulfur Oxidation by New and Non-Canonical Bacteria in a Subsurface Flow Constructed Wetland Treating Domestic Wastewater

**DOI:** 10.3390/microorganisms14030565

**Published:** 2026-03-02

**Authors:** Maricela Arteaga-Mejía, Alida Velázquez-Guadalupe, Elizabeth Castillo-Villanueva, Jorge Valdivia-Anistro

**Affiliations:** 1 Carrera de Biología, Facultad de Estudios Superiores Zaragoza, UNAM, Iztapalapa, Ciudad de México 09230, Mexico; 2 Unidad Multidisciplinaria de Investigación Experimental Zaragoza, Facultad de Estudios Superiores Zaragoza, UNAM, Iztapalapa, Ciudad de México 09230, Mexico

**Keywords:** constructed wetlands, sulfur-oxidizing bacteria, cultivable bacteria, wastewater treatment

## Abstract

Constructed wetlands (CW) are a low-cost alternative for wastewater treatment, where microbial communities play a key role in the biotransformation of pollutants, including sulfur compounds. This study reports the identification of cultivable bacteria involved in the sulfur cycle (SC) in a subsurface-flow CW located in Tetipac, Mexico. Water, sediment, and rhizosphere samples were collected during four sampling events and plated on a mineral medium with thiosulfate. Colony-forming units were quantified, and 15 isolates were genetically identified by partial 16S rRNA gene sequencing. Bacterial abundance was higher in the rhizosphere, and the cultivable fraction was dominated by Pseudomonadota, particularly Gammaproteobacteria, accompanied by Bacteroidota and several previously uncultured lineages; genera such as *Achromobacter*, *Chitinophaga*, *Enterobacter*, *Pseudomonas*, *Raoultella* and *Stenotrophomonas* were recovered. Biochemical assays revealed heterogeneous metabolic profiles, with several isolates showing activities comparable to canonical sulfur-oxidizing bacteria (SOB). Most isolates oxidized thiosulfate and a substantial proportion oxidized elemental sulfur, with higher metabolic performance in rhizosphere isolates and a positive association with BOD_5_ removal. Overall, these results indicate that the Tetipac wetland harbors a cultivable community of largely non-canonical SOB whose mixotrophic versatility and spatial differentiation suggest a key contribution to the SC and organic matter degradation in CW.

## 1. Introduction

Constructed wetlands are widely implemented as cost-effective systems for the treatment of domestic and industrial wastewater, where microbial communities drive the degradation, transformation and removal of pollutants [1,2,3]. Among the relevant biogeochemical processes occurring in these systems, the sulfur cycle is of particular interest because reduced sulfur compounds (e.g., sulfide or thiosulfate) can cause odors, corrosion, metal mobilization and toxicity if not efficiently oxidized or precipitated [4,5,6]. Although the role of sulfur-transforming bacteria has been extensively documented in natural sediments and marine ecosystems [7,8,9], their diversity and activity in constructed wetlands remain poorly characterized, particularly in Latin America [10].

Sulfur oxidation is mainly attributed to well-known chemolithoautotrophic groups such as *Thiobacillus*, *Paracoccus* and *Sulfurimonas*, which use sulfide, thiosulfate or elemental sulfur as electron donors under oxic or microoxic conditions [11,12]. More recently, phylogenetically diverse bacteria have been shown to mediate similar processes through alternative enzymatic pathways or genetic determinants [13,14]. These “non-canonical” sulfur-oxidizing bacteria (SOB) include taxa not traditionally associated with sulfur metabolism, indicating that the capacity for sulfur oxidation is more widespread among heterotrophic and facultatively chemolithotrophic microorganisms than previously recognized [15]. Their metabolic flexibility may contribute to maintaining redox balance and detoxifying reduced sulfur compounds in dynamic environments such as constructed wetlands [16].

Investigating the occurrence and function of both canonical and non-canonical SOB in engineered natural systems is essential to clarify their ecological roles and potential applications. In subsurface flow constructed wetlands, these bacteria can influence sulfur transformations and coupled biogeochemical processes, including nitrogen cycling and metal immobilization [16,17]. Reduced sulfur species and their oxidation products also interact with trace metal biogeochemistry and overall treatment performance, emphasizing the need to identify key sulfur-transforming microbiota in these systems [5,6].

Most studies addressing microbial communities in wetlands rely on culture-independent methods such as high-throughput sequencing and functional gene surveys [18,19,20], whereas culture-based approaches remain valuable to link metabolic traits with phylogeny and to obtain strains for future biotechnological applications [21,22,23]. This study aimed to characterize the physicochemical conditions and diversity of cultivable bacteria involved in the sulfur cycle in a subsurface flow constructed wetland treating domestic wastewater in Tetipac, Mexico. Additionally, the study sought to evaluate the metabolic potential of these bacteria and its relationship with the system’s performance in removing organic matter. The specific objectives of the study were to: (i) conduct a physicochemical characterization of the wetland, (ii) quantify the abundance of cultivable bacteria in water, sediment, and the rhizosphere, (iii) identify representative isolates by 16S rRNA gene sequencing, (iv) describe the taxonomic composition and phylogenetic affiliation of culturable bacterial isolates, and (v) assess their metabolic potential and functional links with wetland performance. This work provides the first culture-based insight into sulfur-transforming microbiota in this treatment system and highlights their possible contribution to pollutant removal in a Latin American constructed wetland.

## 2. Materials and Methods

### 2.1. Study Site and Sampling

**Figure 1:**
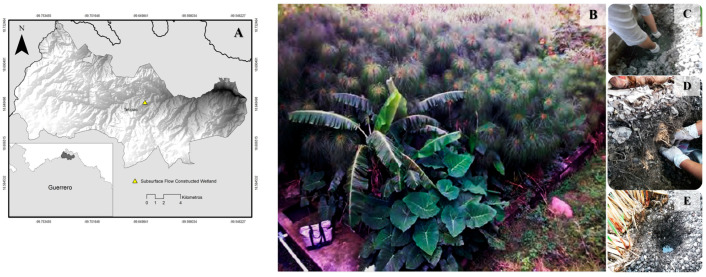
Location (**A**, yellow triangle) and appearance of the constructed subsurface flow wetland in Tetipac, Guerrero (**B**). The types of samples collected in the wetland were: (**C**) water, (**D**) rhizosphere and (**E**) sediment.

Sampling was conducted in a subsurface flow constructed wetland located in Tetipac, Guerrero, Mexico (datum WGS84, UTM 14Q 431945 m E, 2062271 m N; altitude 1640 m), designed for the treatment of municipal wastewater (Figure 1A,B). Four sampling campaigns were carried out in April, May, June and July 2024 at five points along the water flow path. Three types of samples were collected in triplicate: water, sediment attached to the gravel substrate and rhizosphere (roots with adhering soil) (Figure 1C–E). Samples were transported at 4 °C and processed within 24 h.

### 2.2. Physicochemical Characterization of the Wetland System

In situ measurements included pH, temperature, dissolved oxygen (DO), and biochemical oxygen demand (BOD_5_) at the inlet and outlet. These parameters were measured on-site using a Hanna^®^ HI98195 portable multiparameter meter (Hanna^®^ insttruments, Woonsocket, RI, USA). Standard method 5210 was used to determine BOD [24].

### 2.3. Culture-Based Isolation and Colony-Forming Units Quantification

The bacteria were isolated using a culture medium that contained 6 g/L of sodium thiosulfate (Na_2_S_2_O_3_) as the sole source of sulfur [25]. The remaining components were: 1.22 g of monobasic sodium phosphate (NaH_2_PO_4_), 1.39 g of anhydrous dibasic sodium phosphate (Na_2_HPO_4_), 1 g of ammonium chloride (NH_4_Cl), 0.1 g of magnesium chloride (MgCl_2_), 0.03 g of ferric chloride (FeCl_2_), 0.03 g of calcium chloride (CaCl_2_), 0.03 g of manganese (II) chloride (MnCl_2_), 0.5 g of potassium nitrate (KNO_3_), 1 g of sodium acetate (CH_3_COONa), 2 g of sodium bicarbonate (NaHCO_3_), 100 mg cycloheximide (C_15_H_23_NO_4_) and 20 g of bacteriological agar per 1 L.

Serial dilutions were performed on each sample type until the concentration was equivalent to 10^−5^ with respect to the original samples. Then, 100 μL of the diluted samples were spread onto Petri dishes containing the selective medium for SOB. Each sample was spread in triplicate. Petri dish plates were incubated at 30 °C for 3–5 days. Colony-forming units (CFU) were calculated using the equation:

CFU = (Number of colonies/Volume seeded in mL) × Dilution factor

To ensure an accurate estimate, it is important that there are fewer than 200 colonies on the plate. Representative colonies should be classified into morphotypes based on the colony morphology protocol of the American Society for Microbiology [26].

### 2.4. Genomic DNA Extraction and PCR Amplification of the 16S rRNA Gene

Genomic DNA was extracted using a Tris-EDTA (TE) buffer solution (10 mM Tris-HCl and 1 mM EDTA, pH 8.0) supplemented with proteinase K (14 mg/mL) [27]. A 10 µL loop of fresh bacterial biomass from solid media was suspended in 100 µL of TE buffer with proteinase K. The mixture was then incubated at 56 °C for 2 h. The suspension was heated for 10 min at 100 °C, then centrifuged for 15 s at 14,000 rpm. Finally, the aqueous phase was extracted and stored at −18 °C for future use.

The 16S rRNA gene was amplified using universal primers (27F and 1392R). The conditions for PCR amplification were as follows: 94 °C for 1 min of initial denaturation, 35 cycles of denaturation at 94 °C for 1 min, annealing at 56 °C for 30 s, an extension of 1:30 min at 72 °C and a final extension of 5 min at 72 °C. The PCR products were verified by 1.5% agarose gel electrophoresis. The amplicons were commercially sequenced, and the resulting chromatograms were visualized and reviewed using BioEdit version 7.7.1. The curated sequences were compared to the GenBank database using the Basic Local Alignment Search Tool (BLAST^®^) (https://blast.ncbi.nlm.nih.gov/Blast.cgi; 11 December 2025) from the National Center for Biotechnology Information (NCBI).

### 2.5. Phylogenetic Analysis

The sequences were aligned with reference strains using ClustalW 1.8 [28]. Phylogenetic reconstruction was then performed in MEGA 12 using the maximum likelihood method with 1000 bootstrap replicates [29].

### 2.6. Metabolic Screening of Isolates Using Sulfur and Carbon Substrates

The isolates were tested for their ability to use the following sulfur- or carbon-related substrates: elemental sulfur (S^0^), sodium thiosulfate (Na_2_S_2_O_3_), sodium acetate (CH_3_COONa), pyruvate (C_3_H_4_O_3_), citrate (Na_3_C_6_H_5_O_7_), malate (C_4_H_6_O_5_), aspartate (C_4_H_7_NO_4_), methanol (CH_3_OH), and ethanol (CH_3_CH_2_OH). The culture medium used for bacterial isolation was modified by replacing sulfur and organic carbon sources. Elemental sulfur was added at 1% (*w*/*v*), and sodium thiosulfate was used at 20 mM. In addition, sodium acetate was replaced with the selected organic carbon compounds. To circumvent the possibility of inhibitory effects on growth, each organic carbon source was evaluated at a concentration of 1 mM. All concentrations mentioned are expressed per liter of culture medium. Growth assessment was performed after three days of incubation. Isolates showing no visible growth were re-incubated for up to five days. Those remaining inactive were considered negative for substrate utilization.

### 2.7. Determination of Catalase and Oxidase Enzymatic Activities

Catalase activity in bacterial isolates was determined according to [30]. The assay is designed to detect the enzymatic decomposition of hydrogen peroxide (H_2_O_2_) into water and oxygen. This process is evidenced by the formation of bubbles, which indicates a positive catalase reaction.

The oxidase activity of bacterial isolates was determined using Kovács reagent (1.0% tetramethyl-p-phenylenediamine dihydrochloride in distilled water) following [31]. Fresh bacterial biomass (generated within the last 24 h) was applied to sterile filter paper, and two drops of the reagent were added. The appearance of a purple color within 90 s indicated a positive oxidase reaction.

## 3. Results

### 3.1. Physicochemical Parameters of the Constructed Wetland During the Sampling Period

The constructed wetland exhibited stable environmental conditions throughout the sampling period (Table 1). The pH and temperature measurements at the inlet and outlet of the wetland were consistent and showed no significant differences. Dissolved oxygen levels remained low along the flow path. On average, the BOD_5_ concentration decreased from 447.33 ± 185.33 mg/L at the inlet to 89.91 ± 47.57 mg/L at the outlet, corresponding to a removal efficiency of 80.6 ± 47.57%.

### 3.2. Cultivable Bacterial Abundance Among Wetland Compartments

A total of 150 bacterial colonies were recovered across all sampling data. CFU values were consistently higher in rhizosphere samples (Mean = 22,342 CFU/g; SD = 10,255) than in sediment (Mean = 11,770 CFU/g; SD = 17,623) or water (Mean = 282 CFU/mL; SD = 122) (Table 2). Fifteen morphotypes were distinguished based on colony characteristics, reflecting moderate but detectable diversity within the culturable fraction.

Representative isolates were selected monthly from each sample type based on their relative abundance and subsequently subjected to genetic identification. In April, one isolate from the water, one from the sediment, and two from the rhizosphere were analyzed. In May, one water isolate and two isolates each from the sediment and rhizosphere were selected, while in June and July one water isolate and two rhizosphere isolates were analyzed per month.

### 3.3. Taxonomic Composition of Culturable Bacterial Isolates

The processed 15 amplicons of the 16S rRNA gene were 700 bp in length and were deposited in GenBank (Table S1). Taxonomic assignment of the 15 isolates showed that most sequences belonged to the phylum Pseudomonadota, followed by Bacteroidota. Four isolates were initially classified only as “uncultured bacterium” (Table 3); however, three of them were assigned to the class Gammaproteobacteria and one to Betaproteobacteria.

At the class level, the cultivable bacterial community was dominated by Gammaproteobacteria (n = 12), followed by Betaproteobacteria (n = 2) and Chitinophagia (n = 1). At the genus level, the isolates were distributed among *Achromobacter* (n = 1), *Chitinophaga* (n = 1), *Enterobacter* (n = 1), *Pseudomonas* (n = 3), *Raoultella* (n = 2) and *Stenotrophomonas* (n = 3). Additionally, two uncultivable lineages related to *Stenotrophomonas* and *Ralstonia* were identified. Overall, this taxonomic composition indicates that the culturable community was largely composed of Pseudomonadota, particularly Gammaproteobacteria, accompanied by a moderate representation of heterotrophic and potentially sulfur-associated genera.

### 3.4. Phylogenetic Affiliation of Bacterial Isolates and Links to Environmental Origin

**Figure 2:**
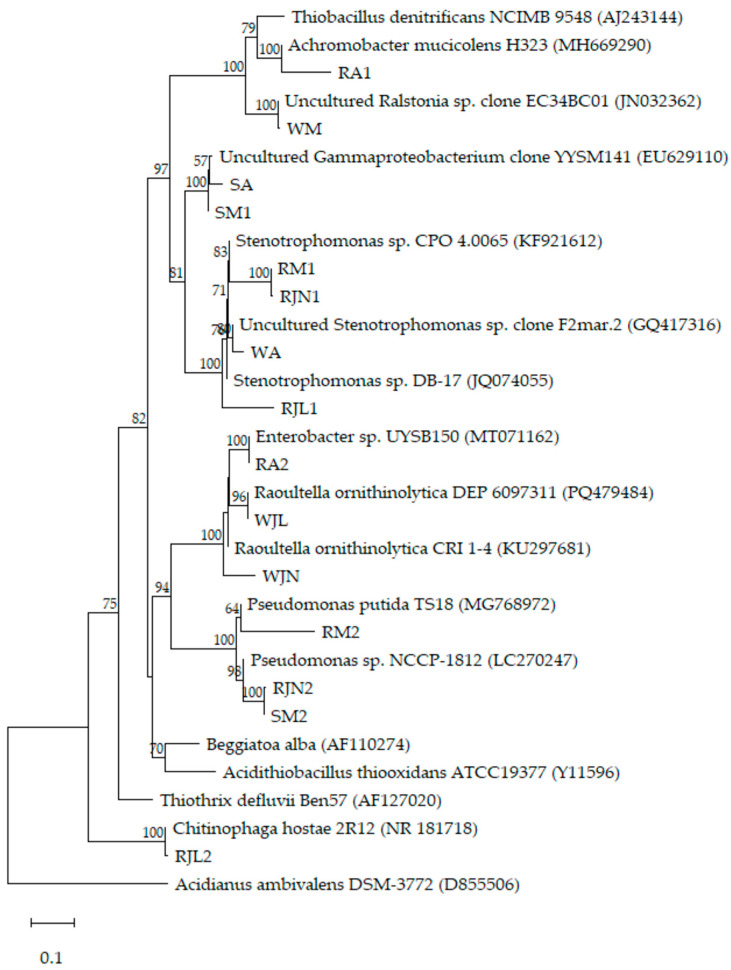
Maximum-likelihood tree based on 16S rRNA gene sequences of the bacterial isolates from the constructed wetland in Tetipac, Guerrero. GenBank accession numbers of the 16S rRNA gene sequences are shown in parentheses. Bootstrap values are expressed as a percentage of 1000 replications. Bar length represents 0.1 substitutions per nucleotide position. The hyperthermophilic archaeon *Acidianus ambivalens* DSM 3772 was used to root the phylogenetic tree.

The phylogenetic assignment of bacterial strains isolated from the different components of the Tetipac constructed wetland revealed a close association between their origin and their environmental functionality (Table 3 and Figure 2). The four bacterial isolates obtained from the water fraction of the wetland showed phylogenetic affinity with strains previously reported in aquatic environments influenced by anthropogenic activities. Isolate WA was found to be closely related to a non-cultivable clone of the genus *Stenotrophomonas* (GenBank accession number GQ417316), exhibiting a sequence similarity of 97.83%. This clone was originally identified in systems associated with the degradation of industrial hydrocarbons. Isolate WM also showed a phylogenetic relationship with an uncultured clone of the genus *Ralstonia* (G.B. acc. no. JN032362), which exhibited a sequence similarity of 99.43% and has been associated with the potential to degrade human urine. Isolates WJN and WJL showed phylogenetic affinity with the genus *Raoultella*, with sequence similarities of 92.61% and 99.71%, respectively. Isolate WJN was found to be associated with a strain obtained from a wastewater treatment constructed wetland (GenBank accession no. KU297681), whereas isolate WJL showed a close relationship to a strain isolated from the effluent of a wastewater treatment plant (GenBank accession no. PQ479484).

The three bacterial isolates obtained from the wetland sediment showed phylogenetic affinity with bacteria associated with terrestrial environments. Isolates SA and SM1 are closely related to an uncultured clone belonging to the class Gammaproteobacteria (GenBank accession no. EU629110), which has been previously detected in forest soil. These isolates exhibit sequence similarities of 99.24% and 99.71%, respectively. In contrast, isolate SM2 showed a 94.19% sequence similarity to an environmental *Pseudomonas* strain characterized by antimicrobial resistance (GenBank accession no. LC270247).

The eight bacterial isolates obtained from the wetland rhizosphere showed phylogenetic affinity with bacteria commonly found in plant-associated environments. In April, two isolates were recovered: RA1 clustered phylogenetically with a root endophytic *Achromobacter mucicolens* strain (GenBank accession no. MH669290), showing 93.87% sequence similarity, whereas RA2 was related to *Enterobacter* sp. UYSB150, a plant growth-promoting bacterium (GenBank accession no. MT071134), with 100% sequence similarity. In May, isolates RM1 and RM2 were affiliated with a rhizosphere *Stenotrophomonas* strain capable of hydrocarbon degradation (91.12% sequence similarity; GenBank accession no. KF921612) and with *Pseudomonas putida* (85.17% sequence similarity; GenBank accession no. MG768972), previously reported as part of the plant microbiome, respectively. The isolates collected in June exhibited a close relationship with two members of the class Gammaproteobacteria: RJN1 showed 90.83% sequence similarity to the same rhizosphere *Stenotrophomonas* strain related to RM1 (GenBank accession no. KF921612), whereas RJN2 showed 93.78% sequence similarity to the same strain related to SM2 (*Pseudomonas* sp. NCCP-1812; GenBank accession no. LC270247). Finally, the isolates collected in July (RJL1 and RJL2) were affiliated with a *Stenotrophomonas* strain isolated from heavy metal-contaminated soil (89.15% sequence similarity; GenBank accession no. JQ074055) and with the rhizosphere bacterium *Chitinophaga hostae* (99.14% sequence similarity; GenBank accession no. NR_181718), respectively.

### 3.5. Metabolic Diversity and Functional Links Between Bacterial Isolates and Wetland Performance

**Figure 3:**
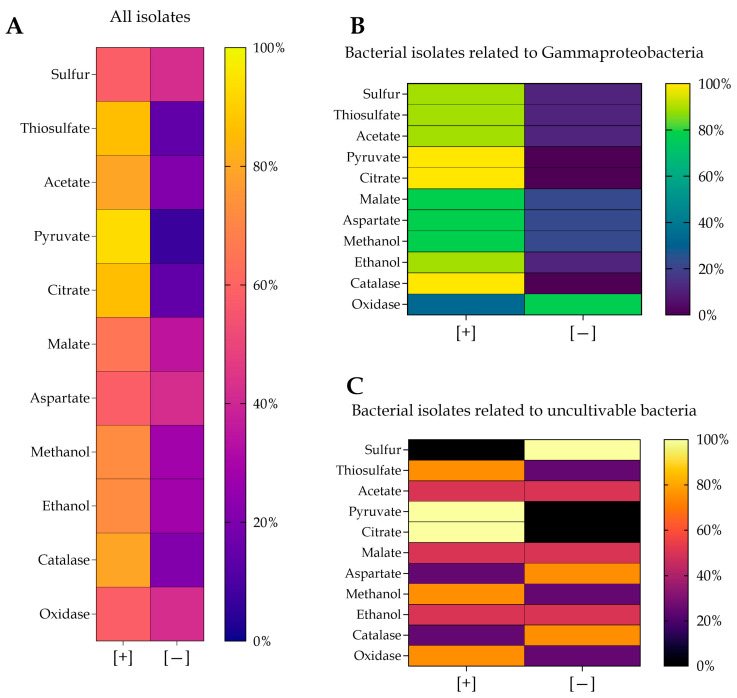
Metabolic capacities of the bacterial isolates from the constructed wetland in Tetipac: (**A**) all the isolates, (**B**) isolates related to Gammaproteobacteria and (**C**) isolates related to uncultured bacteria. [+], positive growth and [−], no growth.

The metabolic capacity of the bacterial isolates from the Tetipac constructed wetland exhibited heterogeneity; however, the majority of isolates demonstrated the capacity to grow on and transform the tested substrates (Figure 3A). The experimental assessment demonstrated that 58% and 86% of the isolates exhibited the capacity to oxidize elemental sulfur and thiosulfate, respectively. For compounds serving as carbon sources, growth was observed in 79% of the isolates with acetate, 93% with pyruvate, 86% with citrate, 65% with malate, and 58% with aspartate. Growth in the presence of methanol and ethanol was 72% in both cases; these two substrates took five days to be metabolized. Finally, 79% of the isolates tested positive for catalase activity and 51% for oxidase activity. According to the two dominant taxonomic groups identified, isolates related to the class Gammaproteobacteria demonstrated optimal growth on all substrates tested; however, 77% of them possess an enzyme other than cytochrome *c* oxidase in the electron transport chain during aerobic respiration (Figure 3B). Conversely, all isolates related to non-cultivable bacteria were unable to grow in the presence of elemental sulfur (Figure 3C). Only 25% of them grew with aspartate and tested positive for catalase, while 50% showed growth in the presence of acetate, malate, and ethanol. Furthermore, 75% of the isolates were found to be positive for thiosulfate, methanol, and oxidase, and 100% of the isolates demonstrated growth in the presence of pyruvate and citrate.

**Figure 4:**
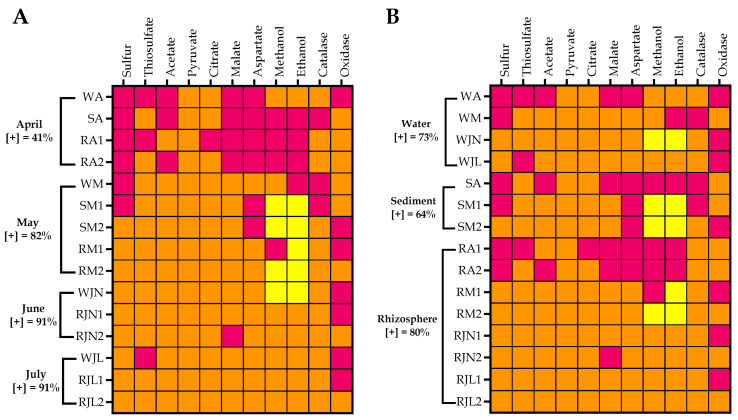
Bacterial metabolic performance by sampling month (**A**) and isolation source (**B**). The meaning of the colors is as follows: orange, positive at 3 days; yellow, positive at 5 days; purple, negative. Sample types: W, water; S, sediment and R, rhizosphere. Sampling month: A, April; M, May; JN, June and JL, July. [+], percentage of bacterial isolates with positive growth.

**Figure 5:**
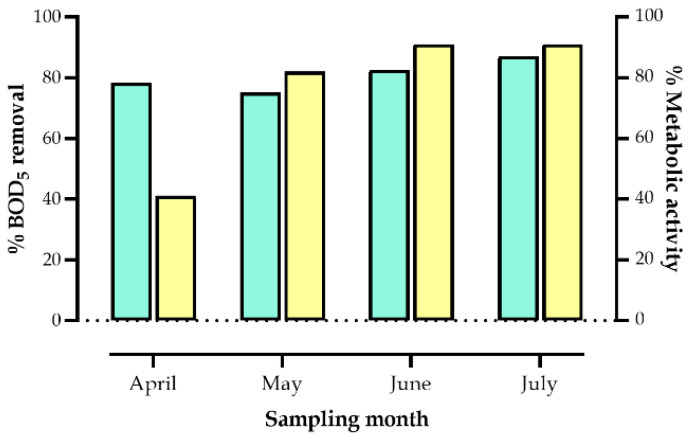
Relationship between the degradation of biodegradable organic matter over 5 days (BOD_5_; green bars) and the metabolic activity of the isolates from the constructed wetland (yellow bars).

The bacterial metabolic performance was related to wetland functionality. This relationship was determined by analyzing the correlation between the metabolic performance and the months of sampling as well as the type of sample isolation. Despite the heterogeneity in the number of isolates selected for analysis, an increase in metabolic performance was observed over time (Figure 4A). The metabolic performance of the isolates was 43% in April, 82% in May, and 91% in June and July. The analysis by sample type showed that rhizosphere isolates exhibited the highest metabolic performance (80%), compared with those from water (73%) and sediment (64%) in the wetland (Figure 4B). With the exception of April, the metabolic performance observed in the bacterial isolates was directly correlated with the degradation of the organic matter entering the wetland (Figure 5). Despite this discrepancy, organic matter removal during that month was 78.3%.

**Figure 6:**
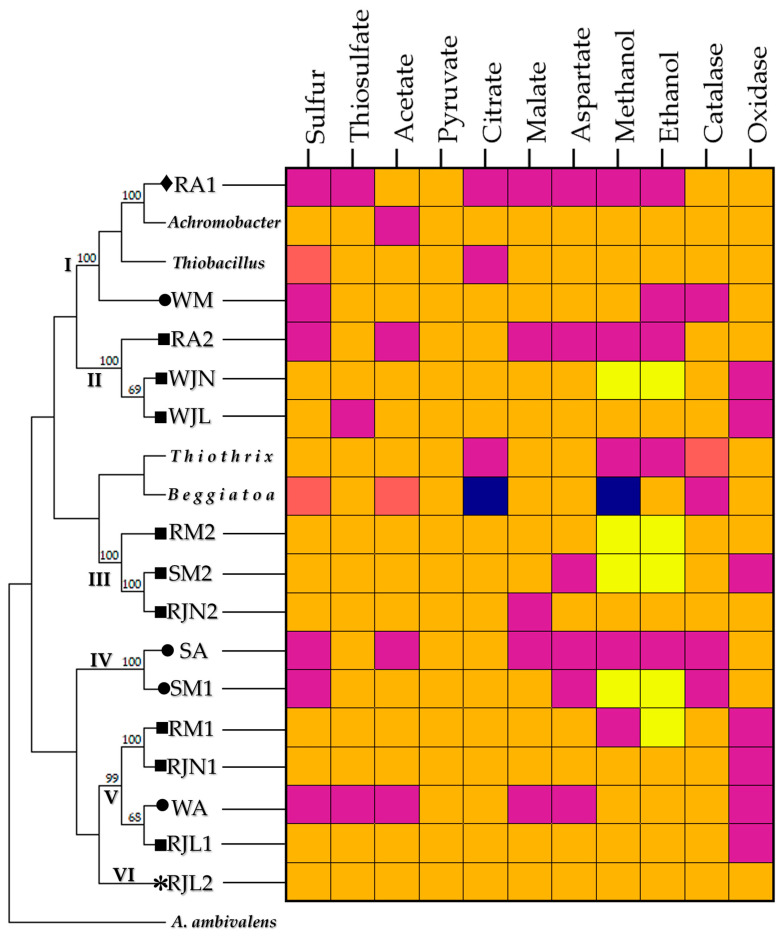
Metabolic profiles of the bacterial isolates from the constructed wetland and the canonical sulfur-oxidizing bacteria. *Achromobacter* was included as a non-traditional sulfur-oxidizing bacterium. Orange, positive at 3 days; yellow, positive at 5 days; purple, negative; pink, variable; blue, not reported in the literature. Symbols indicate taxonomic affiliation: ♦, Betaproteobacteria; ■, Gammaproteobacteria; *, Chitinophagia; ●, uncultured bacteria. *A. ambivalens* was used to root the phylogenetic tree. The Roman numerals represent the groups formed among the bacterial strains isolated from the Tetipac wetland that are phylogenetically related.

To evaluate the relationship between metabolic performance and genetic identity, a maximum-likelihood bootstrap consensus tree was created using 16S rRNA gene sequences from bacterial isolates collected from the Tetipac constructed wetland and reference sulfur-oxidizing bacteria (Figure 6). *Achromobacter* was selected as a reference for a non-traditional sulfur-oxidizing bacterium. The metabolic profiles of the bacteria isolated from the wetland do not match those reported for canonical sulfur-oxidizing bacteria or for *Achromobacter*. Additionally, variations were observed among isolates with identical 16S rRNA gene sequences.

Group I consists of isolates classified as Betaproteobacteria. Isolate RA1 was found to be related to a strain of the genus *Achromobacter*, and it grew exclusively in the presence of acetate and pyruvate. Furthermore, it tested positive in both enzymatic assays. In contrast, isolate WM was related to a non-cultivable clone of the genus *Ralstonia*, showed no growth in the presence of elemental sulfur or ethanol, and tested negative for catalase. Compared with the reference sulfur-oxidizing bacteria, the isolates from the Tetipac wetland demonstrated a more limited metabolic capacity.

Group II consists of three isolates belonging to the Enterobacteriaceae family. Isolate RA2 was found to be related to the genus *Enterobacter* and showed exclusive growth in the presence of thiosulfate, pyruvate, and citrate. Additionally, the results of the enzyme tests were positive. The remaining two isolates, WJN and WJL, are related to strains of *Raoultella ornithinolytica* isolated from wastewater. Both isolates tested negative in the two enzyme tests; isolate WJN reached optimal growth only on the fifth day in the presence of both alcohols, while isolate WJL showed no growth in the presence of thiosulfate.

Three isolates related to the genus *Pseudomonas* constitute group III (Pseudomonadaceae family). This group displayed a notably high level of metabolic efficiency: isolate RM2 showed no negative results in its metabolic characterization, isolate SM2 failed to grow only in the presence of aspartate and tested negative for oxidase, whereas isolate RJN2 did not grow with malate. Isolates RM2 and SM2 both demonstrated sustained growth up to the fifth day in the presence of the two alcohols tested.

Group IV consists of two isolates, both related to a non-cultivable clone of the class Gammaproteobacteria. Neither isolate was able to grow in the presence of elemental sulfur or aspartate, and both tested negative for catalase; however, both were oxidase positive. Isolate SA was able to grow on only three substrates (thiosulfate, pyruvate, and citrate), whereas isolate SM1 exhibited the highest metabolic performance and was characterized by sustained growth up to the fifth day in the presence of both alcohols tested.

Group V consisted of four isolates belonging to the genus Stenotrophomonas (family Xanthomonadaceae). Isolate RM1 demonstrated no growth in the presence of methanol. Isolates RJN1 and RJL1 demonstrated the highest metabolic performance. In contrast, isolate WA was linked to a non-cultivable clone and exhibited the most significant metabolic limitation. All four isolates tested negative for oxidase.

Finally, Group VII contained the isolate RJL2, which is related to the genus *Chitinophaga*. RJL2 tested positive in all metabolic assays.

## 4. Discussion

Constructed wetlands replicate the biogeochemical processes of natural wetlands, facilitating wastewater treatment through the interaction of key components such as the substrate, vegetation, and the microbial community established within the rhizosphere and other system compartments [32,33]. Subsurface flow wetlands require minimal resources for operation and maintenance and are effective in reducing suspended solids, as well as biochemical and chemical oxygen demand [34,35]. Moreover, they limit the proliferation of pathogens and mosquitoes, minimize odor emissions, and contribute to the removal of emerging contaminants and potentially toxic elements [6,36,37]. The physicochemical dynamics observed in the subsurface flow constructed wetland at Tetipac indicate that the prevailing conditions are favorable for sulfur-transforming bacteria, as both oxidizing and reducing microenvironments can coexist within the gravel matrix and the rhizosphere, supporting diverse metabolic activities [5,38].

The pH values measured in the Tetipac constructed wetland are slightly lower than the average reported for other subsurface flow wetlands (pH ~7.6) [39,40]. However, the pH values in Tetipac indicate the neutralizing effect generated by rhizosphere exudates, a condition that favors denitrification, a process associated with sulfur oxidation during wastewater treatment [41,42]. Additionally, the pH of the Tetipac wetland exhibited a decline during the months with the most significant degradation of organic matter, indicative of an accumulation of protons resulting from its oxidation [39]. The dissolved oxygen concentrations in the influent and effluent of the Tetipac wetland fall within the ranges reported for other subsurface flow wetlands [43]. In these systems, dissolved oxygen concentrations are typically low due to water temperature, its consumption during the oxidation of organic matter, and the continuous operation of the wetland [44,45]. In subsurface flow constructed wetlands, influent BOD_5_ can range from 150 to 850 mg/L, while effluent values can be reduced to below 30 mg/L, corresponding to removal efficiencies of 75% to 90% [46,47]. The BOD_5_ values and biodegradable organic matter removal observed in the Tetipac wetland confirm its effectiveness in treating the wastewater it receives, which depends on the bacterial diversity established throughout its structure and components.

The quantification of colony-forming units (CFU) is a measure of the capacity of constructed wetlands to promote microbial activity associated with wastewater treatment [48]. The observed CFU values are consistent with those reported in subsurface flow wetlands and are associated with physicochemical gradients generated as water passes through the microbial matrix of the substrate and rhizosphere [49,50]. The high bacterial abundance measured in the roots is attributable to the fact that the rhizosphere represents an ecosystem distinguished by its elevated microbial diversity and activity [51,52]. Therefore, the higher abundance of CFU in the rhizosphere of the Tetipac wetland indicates that the roots act as microbial hotspots, favored by the release of oxygen and organic exudates, which stimulate biogeochemical oxidation and precipitation processes of organic and inorganic compounds present in the wastewater [53,54].

The taxonomic composition of the isolates recovered from the Tetipac constructed wetland indicates that the culturable community is dominated by members of the phylum Pseudomonadota, particularly Gammaproteobacteria. This pattern is consistent with observations in constructed wetlands, where phyla such as Proteobacteria domain functional microbial assemblages in pollutant-removal systems [20,21]. In subsurface-flow wetlands, the presence of heterogeneous microhabitats promotes the selection of metabolically versatile and fast-growing bacteria, such as Gammaproteobacteria, which can readily adapt to alternating oxidation-reduction conditions [55]. Consequently, Gammaproteobacteria frequently dominate the bacterial assemblages inhabiting wetland sediments and plant roots [56,57]. Genera such as *Enterobacter*, *Pseudomonas* and *Stenotrophomonas* are frequently found at the root-substrate interface of constructed wetlands [22,58,59]. Among these genera, *Pseudomonas* is the only one directly involved in sulfur oxidation in constructed wetlands [16] and is therefore classified as a non-traditional, colorless sulfur-oxidizing bacterium [6]. Furthermore, strains from these genera have been shown to contribute to nitrogen and phosphorus removal (*Pseudomonas*), heavy metal absorption (*Pseudomonas*), and antibiotic degradation (*Pseudomonas* and *Stenotrophomonas*) in constructed wetlands [21,52]. Their prevalence indicates that these taxa may play key roles in organic matter degradation and nutrient cycling, including sulfur compounds, an assertion consistent with the functional importance of Proteobacteria in constructed wetland systems [16,56]. The genus *Raoultella* is noteworthy for its lack of prior documentation as a component of the microbiota associated with constructed wetlands. However, recent reports have identified its presence in wastewater from hospitals, industrial facilities, and swine farms [60,61]. In the present study, the two *Raoultella*-related strains recovered from Tetipac are consistent with this environmental association. The detection of *Raoultella ornithinolytica* in the wetland water is of particular interest. This species has the capacity to remove nitrogen and phosphorus, withstand elevated concentrations of potentially toxic elements, and synthesize phytohormones [62,63]. *R. ornithinolytica* has been proposed as an environmentally friendly candidate for remediation and detoxification processes due to these traits [60,64]. A detailed functional evaluation is necessary to determine the potential capabilities of the two *Raoultella* strains isolated from the Tetipac constructed wetland.

In constructed wetlands, the class Betaproteobacteria is typically represented by bacteria that play central roles in nitrogen transformations [16,21]. One isolate belonging to this class and genetically related to the genus *Achromobacter* was recovered from the rhizosphere of the Tetipac artificial wetland. The genus *Achromobacter* comprises opportunistic pathogens that exhibit multidrug resistance [65]. However, the type species of the genus (*A. xylosoxidans*) is also a sulfur-oxidizing bacterium that has been isolated from rhizosphere and waste-stabilization ponds [66,67]. Due to this metabolic capacity, *Achromobacter* strains are part of the microbial community of constructed wetlands for the treatment of mining leachates [68]. In other constructed wetlands systems, strains of this genus have also been reported to perform denitrification [69,70]. In the present study, the isolate was found to be closely related to the strain *A. mucicolens* H323, a bacterium known to colonize the inner tissues of pine roots. Previous research indicates that *A. mucicolens* is a rhizosphere-associated species detected in constructed wetlands and that its broad metabolic versatility enables the degradation of various xenobiotic compounds [71,72].

The phylum Bacteroidetes is the second most common bacterial group reported in constructed wetlands. In these environments, its members play key roles in the degradation of polymeric organic matter, nitrogen removal, heavy-metal absorption, and antibiotic degradation [16,21,56]. In this phylum, the genus *Chitinophaga* plays a direct role in the ammonification process and the removal of contaminating compounds [69,73]. In the Tetipac constructed wetland, one isolate exhibited a high degree of genetic similarity to *Chitinophaga hostae* 2R12, a species initially described from the rhizosphere of *Hosta plantaginea* [74]. This plant species has been used in constructed wetlands for wastewater treatment [75] and in urban wetlands to improve microclimatic conditions and landscape esthetics [76]. This finding is particularly noteworthy because *H. plantaginea* is not part of the plant community in the Tetipac wetland. This suggests that the presence of this *Chitinophaga* strain reflects broader ecological distribution patterns within the rhizosphere.

Uncultivable bacteria constitute what is referred to as “microbial dark matter”, which represents most of the microbial diversity that cannot be obtained using conventional cultivation techniques [77,78]. The discovery and preliminary characterization of this microbial fraction have been enabled primarily by culture-independent approaches, particularly environmental sequence analysis [79,80]. These studies have revealed not only the enormous extent of yet-unknown bacterial diversity, but also the presence of functional genes associated with novel metabolic pathways, biogeochemical processes, and the biosynthesis of bioactive compounds with potential environmental and biomedical relevance [81,82,83]. Despite the development of advanced experimental strategies to cultivate members of this elusive fraction, culture-dependent methods remain the standard strategy for formal isolation of new bacterial linages [84,85]. In the present study, 28% of the isolates corresponded to bacteria that had not been previously cultured. Multiple culture-independent surveys conducted in constructed wetlands have indicated that the uncultivable bacterial fraction may comprise between 50% and 80% of the total microbial diversity in these systems [86,87,88]. Furthermore, similar taxonomic profiles to those observed in the Tetipac wetland have been reported. Proteobacteria is typically the dominant phylum, followed by uncultured bacterial lineages [87,89]. It is common for uncultivable bacteria to be genetically resolved at different taxonomic depths [90]. In the case of the Tetipac wetland, two of the isolates analyzed (SA and SM1) were assigned to uncultivable bacteria at the class level, while two others (WA and WM) were identified at the genus level. Finally, these last two isolates are related to non-cultivable bacteria from environments influenced by anthropogenic discharges.

The 16S rRNA gene is a widely used molecular marker in bacterial evolution and ecology, particularly for inferring phylogenetic relationships among taxa [91,92]. Its effectiveness as a taxonomic marker stems from its ubiquity across prokaryotes and its high functional and structural conservation [93,94]. Consequently, variation in 16S rRNA gene sequences has long served as the foundation for prokaryotic classification [95,96]. Sequence analysis of this gene remains a standard genotypic criterion in bacterial taxonomy [92]. The established threshold for identifying a novel bacterial species is a minimum of 97% [97]. More recent cut-off values of 95% and 98.7% are commonly applied to delineate genera and species, respectively [98]. Using these taxonomic criteria, six bacterial isolates from the Tetipac wetland were identified at the species level, as they exhibited sequence similarities of over 98.7%. Three of these isolates were affiliated with uncultured bacteria: two were assigned to the class Gammaproteobacteria and one to the genus *Ralstonia*. The other three bacterial isolates were found to be genetically similar to *Chitinophaga hostae* (NR_181718), *Enterobacter* sp. UYSB150 (MT071134) and *Raoultella ornithinolytica* (PQ479484). Furthermore, one isolate obtained from the wetland water was identified at the species level by applying the classical threshold proposed by [97]. This isolate (WA) exhibited 97.83% sequence similarity. However, it is crucial to acknowledge the affiliation of isolated WA with an uncultured clone of the genus *Stenotrophomonas* (GQ417316). The successful isolation of uncultured bacteria represents is a significant contribution to bridging the gap between environmental sequencing data and cultivable microbial diversity.

A notable finding of this study is that 56% of the total isolates recovered from the Tetipac wetland may correspond to novel taxonomic groups. According to the established criteria based on 16S rRNA gene sequence similarity, values between 80% and 90% are indicative of a new family, while values above 90% but below 95% may correspond to a new bacterial genus [99,100,101]. It is noteworthy that six of these isolates were recovered from the wetland rhizosphere. Environments characterized by water-solid interfaces, such as the rhizosphere of constructed wetlands, commonly harbor a high proportion of 16S rRNA gene sequences with similarities below 95%, reflecting the presence of poorly characterized bacterial lineages [102]. Given the importance of rhizosphere bacteria in plant adaptation to multiple stress factors, there is increasing interest in isolating bacterial representatives that have not yet been cultivated [103,104]. The genetic identity levels of previously uncultured bacteria that have been successfully isolated typically range from 80% to 95%, enabling the recovery of novel microorganisms with potentially new metabolic functions [105,106]. In general, uncultured taxa that are successfully isolated predominantly belong to the phylum Proteobacteria [107], a pattern consistent with the findings of the present study. The results of this study align with those reported for other constructed wetlands [108,109,110], which are recognized as significant sources for the discovery of novel bacterial species with potentially innovative metabolic functions applicable to bioremediation processes [111,112,113]. However, the affiliations proposed for the uncultured bacteria isolated from the Tetipac constructed wetland should be confirmed through a polyphasic taxonomic approach [95,114,115].

Metabolic screening revealed clear differences in substrate utilization patterns among the bacterial strains isolated from water, sediment, and the rhizosphere. This metabolic heterogeneity was related to climatic seasonality: in June and July, the highest removal of biodegradable organic matter and the best bacterial metabolic performance in the rhizosphere were observed. In Tetipac and the surrounding areas, these months correspond to the late spring and the onset of the rainy season, a period during which constructed wetlands typically operate optimally in the removal of organic matter and contaminant compounds [116,117,118]. Furthermore, the rhizosphere of constructed wetlands is the primary zone where the purification processes of these substances occur [119,120]. These results suggest that the combined effect of climatic seasonality and rhizosphere processes is a key factor regulating bacterial metabolic potential and, consequently, the efficiency of constructed wetlands in removing organic matter and contaminants.

The cultivable bacterial diversity in the Tetipac constructed wetland was dominated by the class Gammaproteobacteria. This bacterial class, distinguished by its high metabolic versatility and strong adaptive capacity, often colonizes the rhizosphere of constructed wetlands employed for wastewater treatment [20,55,57]. Research has shown that environmental Gammaproteobacteria possess genes involved in the oxidation and reduction in different sulfur compounds (mainly elemental sulfur, sulfate, and thiosulfate). These genes are associated with enzymatic complexes that facilitate the degradation of various organic compounds [121,122]. Furthermore, this bacterial class demonstrates a high tolerance to xenobiotic compounds, which subsequently enables them to develop the capacity to degrade and utilize these compounds as metabolic resources [123,124]. This metabolic potential has also been observed in Gammaproteobacteria that have not yet been cultivated [125,126]. The prevalence of Gammaproteobacteria and their potential involvement in the metabolism of sulfur compounds, as well as in the degradation and utilization of carbohydrates and xenobiotic compounds, has been documented in both natural and constructed wetlands [20,127]. Consequently, the high abundance of this class in the Tetipac constructed wetland is consistent with its well-recognized metabolic versatility.

Bacteria not yet cultivated constituted the second most abundant group identified in the Tetipac constructed wetland. This group represents one of the most diverse and dominant fractions in natural environments and acts as a reservoir of novel metabolic capabilities with potential applications in biotechnological and bioremediation processes [77,81,83]. Other studies have shown that, after Proteobacteria, uncultivable bacteria are the most abundant group in constructed wetlands [87,89]. In Tetipac, 75% of the uncultivated bacteria isolated were genetically related to the class Gammaproteobacteria, which includes representatives within this unknown fraction of bacterial diversity that exhibit unique metabolic properties [125,126]. This could explain the low metabolic performance observed for the chemical forms evaluated in the present study. In general, uncultivable bacteria display specific nutritional and growth requirements that make their isolation difficult under laboratory conditions [128,129,130]. Despite the growing interest in quantifying bacterial diversity using culture-independent methods, the isolation and laboratory cultivation of new bacteria remains the only strategy that allows for detailed characterization of their metabolic potential and its exploitation to develop biotechnological alternatives to address environmental and public health problems.

Growth-related properties are an integral part of bacterial phenotypic characterization, facilitating their accurate description and differentiation [131,132]. Growth phenotypes are a useful starting point for understanding the evolutionary factors that influence whether a bacterium can develop in a broad or restricted environment [133,134]. At the metabolic level, bacteria first evolve to exploit basic and abundant elemental nutrients and subsequently adapt to counteract the harmful effects of environmental stress factors and toxic substances [135,136]. For all these reasons, the characterization of metabolic profiles is a key component in the classification and proposal of new bacterial species [95,114]. The metabolic profiles of the bacteria isolated from the wetland differed from those previously reported for both canonical and non-canonical sulfur-oxidizing bacteria. In addition, metabolic variation was observed even among isolates that shared identical genetic identities.

Four bacteria isolated from the Tetipac constructed wetland were affiliated with the genus *Stenotrophomonas*: one from the water and three from the rhizosphere. The genus *Stenotrophomonas*, classified within the class Gammaproteobacteria, is distinguished by its ability to undergo aerobic growth and by its presence in the rhizosphere, where it acquires the sulfur necessary for synthesizing sulfur-containing amino acids [137]. Additionally, it has been observed to metabolize acetate, citrate, malate, and pyruvate, yet it is unable to utilize aspartate [138]. Furthermore, a positive catalase reaction and a negative oxidase reaction have been noted [137]. The rhizosphere isolates from the Tetipac wetland generally conform to the properties described for the genus and are notable for their ability to metabolize thiosulfate, aspartate, methanol, and ethanol. It has been demonstrated that the metabolism of thiosulfate is prevalent among *Stenotrophomonas* strains found in the rhizosphere [67]. In addition to establishing themselves to obtain sulfur chemical forms, members of this genus have been shown to increase sulfate availability in the rhizosphere, thereby promoting plant growth [139]. To date, there have been no reports of *Stenotrophomonas* strains capable of metabolizing methanol or ethanol. The only alcohol described as a utilizable substrate for this genus is *n*-propanol [137]. *Stenotrophomonas* does not require aspartate as an essential amino acid. However, rose essential oil’s antimicrobial effect has been observed to increase aspartate content. This increase has been attributed to changes in bacterial cell membrane permeability and to a metabolic imbalance that induces atypical activities [140]. These alterations hypothetically suggest that aspartate could be exploited as an alternative carbon source or for protein synthesis under stress conditions [141]. In addition, *Stenotrophomonas* has been identified in wastewater, where it plays a role in the oxidation of sulfur compounds. This ability, when combined with other bacteria, contributes to the efficient removal of nitrate [142,143]. However, the strain isolated from the wetland water demonstrated the most limited metabolic repertoire, which could be explained by its relationship to a non-cultivable clone of the genus. This clone may possess other, as yet uncharacterized, metabolic capacities.

Two bacterial isolates obtained from the wetland sediment clustered with an uncultivated clone belonging to the Gammaproteobacteria class. The isolate collected in April sampling displayed the strongest metabolic limitation. The Tetipac wetland isolates with restricted metabolic repertoires are likely related to uncultivable bacteria whose ecology relies more on tight metabolic interactions within the microbial community than on a broad individual capacity to use multiple carbon and energy sources [144,145,146]. The observed limitation in metabolic capacity in isolates related to uncultivable bacteria does not represent a generalized characteristic. The other isolate, collected in May, failed to grow only in the presence of elemental sulfur and aspartate. Several environmental genomics studies have shown that many uncultivable bacteria have reduced genomes and incomplete metabolic pathways [147,148,149]. This translates into a limited ability to synthesize essential compounds and to utilize a wide range of substrates. Taken together, these results suggest that metabolic limitation is not solely explained by affiliation with uncultivable lineages, but rather, it is a trait that can arise in bacteria that depend on community-level interactions and in strains with broader yet constrained metabolic capacities. These considerations help to explain the observed heterogeneity in metabolic capabilities among all bacterial isolates from the Tetipac wetland related to uncultivable bacteria. However, metabolic limitation does not appear to be an exclusive trait of uncultivable bacteria.

A bacterial isolate obtained from the rhizosphere of the Tetipac wetland was genetically related to the *Achromobacter mucicolens* strain, which is described as a pine root endophyte. The isolate could not grow in the presence of elemental sulfur or thiosulfate; it metabolized only acetate and pyruvate and tested positive in both enzymatic assays. The type species of the genus, *A. xylosoxidans*, along with other environmental strains, tests positive in all the assays evaluated in this study, with the exception of acetate [67,150,151]. Studies of experimental evolution and comparative genomics have demonstrated that targeted losses or reductions in metabolic functions can occur even in culturable bacteria, resulting in more restricted yet adaptive catabolic repertoire under specific environmental conditions [152,153]. Under copiotroph conditions or in communities where certain metabolites, such as amino acids or aromatic compounds, are available, bacteria that lose biosynthetic or degradative pathways and depend on external resources or cross-feeding from other strains have been selected [154,155]. This reduces cellular costs at the expense of metabolic versatility. These findings support the hypothesis that the metabolic limitations observed in some wetland isolates are not artifacts of isolation but rather outcomes of evolutionary processes of specialization and metabolic dependency [156,157].

The remaining bacterial isolates from the Tetipac wetland displayed metabolic variability consistent with previously described patterns. Notably, some isolates exhibited distinctive metabolic traits or unique combinations resembling those characteristics of the genus *Stenotrophomonas*. The cosmopolitan genus *Ralstonia* comprises species inhabiting aquatic and rhizospheric environments that metabolize citrate, malate, aspartate, and ethanol, and are catalase- and oxidase-positive [158]. The isolate obtained from Tetipac water was found to be phylogenetically related to a non-cultivable clone of this genus, which had been previously detected in a cave contaminated with urine. While this isolate did not oxidize elemental sulfur, environmental *Ralstonia* strains from mining leachates have been reported to participate in sulfur oxidation [159]. Sulfur oxidation in this genus may yield thiosulfate, which can subsequently be reduced during anaerobic ammonium oxidation [160]. In wastewater systems, *Ralstonia* strains have been found to produce ethanol from acetate and further transform it into polyhydroxybutyrate [161]. Although there have been no reports of pyruvate and methanol metabolism for this genus, pyruvate has been shown to stimulate the growth of viable but non-cultivable *Ralstonia* strain [162], which may explain the isolate obtained in this study. Furthermore, *R. pickettii* has been observed to acquire genes via horizontal transfer under extreme aquatic conditions, thereby expanding its range of carbon utilization [163]. This phenomenon may provide a biological explanation for the methanol metabolism observed in the Tetipac isolate.

The genera *Enterobacter* is frequently detected in the rhizosphere of subsurface flow constructed wetlands [59]. In this wetland compartment, it participates in phosphate removal, drug degradation, and the accumulation or uptake of heavy metals [21,22,164]. The genus *Enterobacter* comprises facultative anaerobes capable of metabolizing acetate, citrate, malate, and aspartate [165]. In the genus description, it is noted that they do not produce hydrogen sulfide from thiosulfate. The isolate examined in this study was found to be related to a plant growth–promoting strain. However, it demonstrated growth in the presence of thiosulfate and pyruvate, and tested positive for both catalase and oxidase. In newly described *Enterobacter* species isolated from the rhizosphere and its surrounding environment, growth has been observed in the presence of pyruvate but not in the presence of thiosulfate or alcohols, and only one of these species tests positive for catalase [166,167,168]. Research has shown that only one *E. hormaechei* strain isolated from manure has demonstrated the capacity to oxidize thiosulfate [169]. However, the complete sulfur metabolism pathway has been identified in the genome of a root endophytic strain, along with the ability to promote plant growth and modulate the expression of the sulfur regulon in its host plant [170]. Overall, the genus Enterobacter plays distinct yet complementary roles in the rhizospheric microbiota of constructed wetlands. *Enterobacter* species play a crucial role in nutrient cycling and plant–microbe interactions due to their versatile metabolic capacity and plant growth–promoting potential. This contributes to the functional resilience of wetland microbial communities. This genus is integral to the biogeochemical network that underpins the removal of pollutants and the maintenance of ecological stability in subsurface flow constructed wetlands.

The genus *Pseudomonas* is among the most prevalent in the rhizosphere of constructed wetlands. In addition to contributing to pharmaceutical degradation, it has been shown to reduce sulfur to sulfides and utilize various organic compounds, such as sugars, acetate, and ethanol, as electron donors for sulfur reduction [171,172]. In this study, three isolates related to the genus *Pseudomonas* were obtained from the rhizosphere and sediment and exhibited minor metabolic variations, a trait commonly observed among closely related species with similar physiological potential [173]. In contrast to the findings reported for other constructed wetlands, the *Pseudomonas* isolates from Tetipac demonstrated the ability to oxidize sulfur and grow on the tested compounds. The sulfur-oxidizing ability observed in these isolates highlights the ecological versatility of *Pseudomonas* in constructed wetlands. This suggests a potential adaptive role in sulfur turnover under fluctuating redox conditions, supporting its emerging recognition as a nontraditional sulfur-oxidizing taxon [6,16].

Bacteria involved in the sulfur cycle constitute a specialized ecological guild [174,175]. The oxidation of reduced sulfur compounds is carried out by a broad phylogenetic and physiological diversity of chemolithoautotrophic bacteria, characterized by remarkable environmental adaptability and versatility [11]. In recent years, the study of sulfur-oxidizing bacteria has gained increasing attention due to their potential environmental and technological applications [15,66]. This growing interest stems from their ability to metabolize diverse chemical forms of sulfur under a wide range of environmental conditions [176]. Sulfur is a common chemical component in constructed wetlands and is susceptible to redox transformations coupled to bacterial metabolism, which favors its removal [177]. Most studies on sulfur oxidation in wastewater have focused on bacteria belonging to the canonical microbial guild associated with the biogeochemical sulfur cycle [17]. However, several additional bacterial groups have been shown to mediate comparable oxidative transformations without being traditionally classified within this guild [15]. The presence of bacteria performing equivalent biogeochemical functions is common in constructed wetlands [16]. Our results confirm that sulfur-oxidation capacity is more phylogenetically and functionally widespread than previously recognized. This functional breadth across different bacterial taxa is a consequence of gene flow mediated by horizontal gene transfer, which has enabled biogeochemical processes driven by microbial metabolic activity to be maintained throughout Earth’s geological history [178]. The sulfur cycle is recognized as one of the oldest biogeochemical cycles. The associated bacterial metabolic processes have been examined at the phylogenetic level, revealing that the evolution of this biogeochemical cycle has been intimately linked to Earth’s redox regimes throughout history [179]. The analysis of the evolutionary history of bacterial sulfur oxidation supports this interpretation [13,180]. Furthermore, the study of the genes involved in this oxidation process has led to the development of new research avenues [181]. The sulfur-oxidizing bacteria identified in this study are mixotrophic, meaning they can metabolize different organic compounds [15,17,176]. This property has the potential to enhance the treatment of wastewater entering the Tetipac wetland. In addition, the bacterial genera identified in Tetipac has been reported to degrade antibiotics in other constructed wetlands [21,52,126]. This property must be experimentally confirmed in this system, and it is highly relevant given the increased abundance of these compounds in wastewater since the COVD-19 pandemic [182]. Therefore, the sulfur-oxidizing bacteria identified in the Tetipac constructed wetland can be considered part of the non-canonical microbial fraction of the sulfur cycle, and their genetic identity provides partial verification of the functional roles they perform in this system. This study makes a valuable contribution to our understanding of the cultivable fraction of the bacterial diversity in this wetland. This diversity represents a significant microbial reservoir with the potential to enhance our capacity to treat wastewater entering this system.

## 5. Conclusions

The Tetipac subsurface flow constructed wetland is home to a diverse and adaptable bacterial community that is structured by rhizosphere–substrate microhabitats and seasonal dynamics. Together, these factors support efficient removal of organic matter and sulfur compounds. Our culture-based analysis revealed the dominance of Gammaproteobacteria, the presence of non-canonical mixotrophic sulfur-oxidizing taxa and previously uncultured lineages, and marked metabolic differentiation among isolates from water, sediment, and roots. Furthermore, more than half of the isolates exhibited 16S rRNA gene similarity values indicative of putatively novel taxa, and several lineages previously detected only as uncultured clones were successfully cultivated under wetland-derived conditions. This study combines detailed metabolic profiling with phylogenetic resolution at the species and higher taxonomic levels. As a result, it uncovers non-canonical sulfur oxidation and substrate-use patterns in genera such as *Enterobacter*, *Raoultella*, *Stenotrophomonas*, and *Achromobacter*. These patterns have not been reported previously in constructed wetlands. These findings indicate that constructed wetlands not only act as efficient wastewater treatment systems, but also represent reservoirs of poorly characterized bacteria with previously underappreciated biogeochemical functions and promising biotechnological potential, including the degradation of emerging contaminants such as antibiotics.

## Figures and Tables

**Table 1:** Physicochemical parameters recorded in the constructed wetland during the sampling period. The values of dissolved oxygen and BOD_5_ are in milligrams per liter (mg/L). SD = standard deviation.

	pH		Temperature (°C)		Dissolved Oxygen		BOD_5_		BOD_5_ Removal
Sampling Month	Inlet	Outlet	Inlet	Outlet	Inlet	Outlet	Inlet	Outlet	
April	6.82	7.02	24.3	24.5	1.21	1.82	765.69	166.44	78.3
May	6.96	7.09	23.3	23.3	2.02	1.41	359.7	89.7	75.1
June	7.09	6.96	22.01	20.5	0.51	0.46	363.95	63.71	82.5
July	6.8	6.4	21	23	1.01	0.6	300	39.8	87.0
Mean ± SD	6.92 ± 0.12	6.87 ± 0.27	22.65 ± 1.25	22.82 ± 1.45	1.19 ± 0.54	1.07 ± 0.56	447.33 ± 185.53	89.91 ± 47.57	80.6 ± 4.4

**Table 2:** Abundance of cultivable bacteria (CFU) in water, sediment, and rhizosphere samples. NG = no growth of bacterial colonies.

Sampling Month	Water (CFU/mL)	Sediment (CFU/g)	Rhizosphere (CFU/g)	Number of Morphotypes
April	184	4990	5650	5
May	233	42,090	33,200	8
June	492	NG	23,370	3
July	221	NG	27,150	6
Mean ± SD	282 ± 122	11,770 ± 17,623	22,342 ± 10,255	

**Table 3:** 16S rRNA gene-based identification of selected isolates. Sample types: W, water; S, sediment and R, rhizosphere. Sampling month: A, April; M, May; JN, June and JL, July.

Isolate Code	Class	Closest Match (NCBI BLAST)	Habitat/Function	% Identity
WA	Uncultured bacterium	Uncultured *Stenotrophomonas* sp. clone F2mar.2 (GQ417316)	Biological degreasing system	97.83
SA		Uncultured Gammaproteobacterium clone YYSM141 (EU629110)	Forest soil	99.24
RA1	Betaproteobacteria	*Achromobacter mucicolens* H323 (MH669290)	Endophytic bacteria of pine tree	93.87
RA2	Gammaproteobacteria	*Enterobacter* sp. UYSB150 (MT071134)	Plant growth-promoting bacteria	100
WM	Uncultured bacterium	Uncultured *Ralstonia* sp. clone EC34BC01 (JN032362)	Cave; urine-degrading bacteria	99.43
SM1		Uncultured Gammaproteobacterium clone YYSM141 (EU629110)	Forest soil	99.71
SM2	Gammaproteobacteria	*Pseudomonas* sp. NCCP-1812 (LC270247)	Environmental samples; antibiotic resistance testing	94.19
RM1		*Stenotrophomonas* sp. CPO 4.0065 (KF921612)	Rhizosphere; hydrocarbon-degrading bacteria	91.12
RM2		*Pseudomonas putida* TS18 (MG768972)	Plant	85.17
WJN		*Raoultella ornithinolytica* CRI 1-4 (KU297681)	Rhizosphere; constructed wetland	92.61
RJN1		*Stenotrophomonas* sp. CPO 4.0065 (KF921612)	Rhizosphere; hydrocarbon-degrading bacteria	90.83
RJN2		*Pseudomonas* sp. NCCP-1812 (LC270247)	Environmental samples; antibiotic resistance testing	93.78
WJL		*Raoultella ornithinolytica* DEP_6097311 (PQ479484)	Wastewater treatment plant	99.71
RJL1		*Stenotrophomonas* sp. DB-17 (JQ074055)	Metal rich soil	89.15
RJL2	Chitinophagia	*Chitinophaga hostae* 2R12 (NR_181718)	Rhizosphere	99.14

## Data Availability

The results of this study have been submitted to the GenBank database under accession numbers: PX908460; PX908461; PX908481; PX908698; PX908699; PX908700; PX908701; PX908702; PX908704; PX908705; PX908706; PX915708; PX915792; PX919758; PX919778.

## Supplementary Materials

The following supporting information can be downloaded at: https://www.mdpi.com/article/10.3390/microorganisms14030565/s1, Table S1: Classification of bacterial isolates from the Tetipac constructed wetland based on 16S rRNA gene sequences.

## Abbreviations

The following abbreviations are used in this manuscript:

CW	Constructed wetland
SC	Sulfur cycle
SOB	Sulfur-oxidizing bacteria
DO	Dissolved oxygen
BOD	Biochemical oxygen demand
CFU	Colony-forming units
