# Supplementary material for: Sulfur Oxidation by New and Non-Canonical Bacteria in a Subsurface Flow Constructed Wetland Treating Domestic Wastewater
Source: Microorganisms. 2026 Mar 2;14(3):565. doi: 10.3390/microorganisms14030565 (PMC13029343; doi:10.3390/microorganisms14030565)
Supplement: Supplementary file 1 [file microorganisms-14-00565-s001.zip › microorganisms-4151649-supplementary.pdf]

Table S1: Classification of bacterial isolates from the Tetipac constructed wetland based on 16S rRNA gene sequences.

Isolate classification	Accession number	Closest match (NCBI BLAST)	% identity
Stenotrophomonas sp. LEMAZ-WA	PX908704	Uncultured *Stenotrophomonas* sp. clone F2mar.2 (GQ417316): Bacterial community composition of biological degreasing systems and health risk assessment for workers (PubMed: 21698403).	97.83
Gammaproteobacteria bacterium LEMAZ-SA	PX908481	Uncultured Gammaproteobacterium clone YYSM141 (EU629110): Bacterial community diversity in undisturbed perhumid montane forest soils in Taiwan (PubMed: 19727930).	99.24
Achromobacter sp. LEMAZ-RA1	PX908460	Betaproteobacteria; *Achromobacter mucicolens* H323 (MH669290): Isolation of bacterial endophytes from pine tree [Unpublished].	93.87
Enterobacter sp. LEMAZ-RA2	PX908706	Gammaproteobacteria; *Enterobacter* sp. UYSB150 (MT071134): Plant growth-promoting bacteria in *Sorghum bicolor* [Unpublished].	100
Ralstonia sp. LEMAZ-WM	PX908700	Uncultured *Ralstonia* sp. clone EC34BC01 (JN032362): The microbiological impact and potential for the bioremediation of human urine in caves [Unpublished]	99.43
Gammaproteobacteria bacterium LEMAZ-SM1	PX908698	Uncultured Gammaproteobacterium clone YYSM141 (EU629110): Bacterial community diversity in undisturbed perhumid montane forest soils in Taiwan (PubMed: 19727930).	99.71
Pseudomonas sp. LEMAZ-SM2	PX915708	Gammaproteobacteria; *Pseudomonas* sp. NCCP-1812 (LC270247): Screening of microbes isolated from environmental samples for antibiotic resistance [Unpublished]	94.19
Lysobacteraceae bacterium LEMAZ-RM1	PX915792	Gammaproteobacteria; *Stenotrophomonas* sp. CPO 4.0065 (KF921612): Hydrocarbonoclastic phylogeny of bacteria associated with the rhizosphere of mangrove [Unpublished].	91.12
Pseudomonadaceae bacterium LEMAZ-RM2	PX908699	Gammaproteobacteria; *Pseudomonas putida* TS18 (MG768972): Plant microbe interaction [Unpublished].	85.17
Raoultella sp. LEMAZ-WJN	PX908702	Gammaproteobacteria; *Raoultella ornithinolytica* strain CRI 1-4 (KU297681): Assessment of *Canna flaccida* culturable endophytic community inhabiting a wastewater treatment constructed wetland [Unpublished]"	92.61
Lysobacteraceae bacterium LEMAZ-RJN1	PX919758	Gammaproteobacteria; *Stenotrophomonas* sp. CPO 4.0065 (KF921612): Hydrocarbonoclastic phylogeny of bacteria associated with the rhizosphere of mangrove [Unpublished].	90.83
Pseudomonas sp. LEMAZ-RJN2	PX919778	Gammaproteobacteria; *Pseudomonas* sp. NCCP-1812 (LC270247): Screening of microbes isolated from environmental samples for antibiotic resistance [Unpublished]	93.78
Raoultella ornithinolytica LEMAZ-WJL	PX908701	Gammaproteobacteria; *Raoultella ornithinolytica* DEP_6097311 (PQ479484): Bacterial isolates obtained	99.71

		from wastewater treatment plant effluent (Ostia) [Unpublished]	
Xanthomonadaceae bacterium LEMAZ-RJL1	PX908705	Gammaproteobacteria; *Stenotrophomonas* sp. DB-17 (JQ074055): Bacteria from metal rich soil [Unpublished].	89.15
Chitinophaga hostae LEMAZ-RJL2	PX908461	Chitinophagia; *Chitinophaga hostae* 2R12 (NR_181718): *Chitinophaga* sp. nov., isolated from isolated from the rhizosphere soil [Unpublished].	99.14
